# ANCA-Positive Small-Vessel Vasculitis Following SARS-CoV-2 Vaccination—A Systematic Review

**DOI:** 10.3390/vaccines12060656

**Published:** 2024-06-13

**Authors:** Kinga Łysak, Agata Walulik, Michał Błaszkiewicz, Krzysztof Gomułka

**Affiliations:** 1 Faculty of Medicine, Medical University of Gdansk, 80-210 Gdansk, Poland; kinga.lysak@op.pl; 2 Student Scientific Group of Internal Medicine and Allergology, Wroclaw Medical University, 50-368 Wroclaw, Poland; 3 Department of Internal Medicine, Pneumology and Allergology, Wroclaw Medical University, 50-368 Wroclaw, Poland; krzysztof.gomulka@umed.wroc.pl

**Keywords:** COVID-19, SARS-CoV-2, vaccine, ANCA-associated vasculitis, MPO-ANCA, PR3-ANCA

## Abstract

As vaccinations against the SARS-CoV-2 virus have become a crucial tool in controlling the spread of the disease, reports of rare health complications have emerged, including new-onset antineutrophil cytoplasmic autoantibodies (ANCA)-associated vasculitis (AAV). We systematically reviewed new-onset AAV following COVID-19 vaccination case reports and case series published in three databases before January 2024 following PRISMA guidelines to understand the characteristics of possible causal relationships or coincidences. In total, 404 articles were screened respectively by title, abstracts, and full-texts. Thirty-four papers fulfilled the inclusion criteria and have been analyzed, covering 44 patients with new-onset AAV after COVID-19 vaccination with no prior history of COVID-19 infection. Data regarding patients’ metrics, comorbidities, vaccination characteristics, symptoms, diagnostics, treatment, and outcomes were investigated and summarized. The cohort consisted predominantly of females. AAV diagnosis was confirmed via biopsy, with renal dysfunction as a prevailing manifestation. In most cases, the first symptoms of AAV developed after the second dose; moreover, Pfizer-BioNTech was the most frequently administered vaccine among the analyzed cohort. Primary treatment involved glucocorticoid therapy, with a mostly favourable response. This systematic review aims to raise awareness among clinicians in the field regarding this rare but possible complication, to promote the prompt recognition and diagnosis of de novo ANCA-positive small-vessel vasculitis in timely association with SARS-CoV-2 vaccination.

## 1. Introduction

Immunization stands as an instrument for global health and development achievements, annually preserving millions of lives worldwide. Vaccines reduce the risks of getting a disease by operating in harmony with the body’s innate defences. Following vaccination, the immune system is prompted to react, thereby establishing a shield of protection [1].

Coronavirus disease 2019 (COVID-19), caused by severe acute respiratory syndrome coronavirus 2 (SARS-CoV-2), has resulted in more than 670 million infections and almost 7 million deaths globally [2]. With the ongoing COVID-19 pandemic and the emergence of new variants of SARS-CoV-2, there is a critical demand for vaccines. These vaccines are essential for safeguarding individuals at elevated risk of complications and potentially controlling disease outbreaks through the establishment of herd immunity [3]. In early 2021, the first vaccines were introduced to stop the pandemic. Also, approximately 68.2% of the world’s population has been fully vaccinated against this disease.

There are four major strategies for producing COVID-19 vaccines, including nucleic, acid-based vaccine (DNA–mRNA), viral vector (replication–non-replication), live inactivated (or attenuated) virus, and protein (spike protein or its subunits) [4].

Vaccination has long been recognized as an integral part of primary care medicine, serving to prevent a wide array of common and potentially life-threatening diseases. However, they can cause common side effects, including injection site pain, redness, and swelling and systemic symptoms such as fever, malaise, headache, and lymphadenopathy. However, it is important to acknowledge the potential for more serious adverse events associated with certain vaccinations [5]. Recently, there have been an increasing number of reports of autoimmune diseases either flaring up or occurring de novo. These include autoimmune glomerulonephritis, autoimmune rheumatic diseases, and autoimmune hepatitis [2]. Furthermore, there is a growing acknowledgement of the occurrence of new-onset antineutrophil cytoplasmic antibody (ANCA)-associated small-vessel vasculitis in connection with SARS-CoV-2 vaccinations [6].

ANCA-associated vasculitis (AAV) is a group of small-vessel vasculitis characterized by the severe inflammation of small blood vessels. This disorder manifests through a breakdown in the body’s tolerance to neutrophil primary granule proteins, such as leukocyte proteinase 3 (PR3) or myeloperoxidase (MPO) [7]. AAV is clinically classified into three main types, granulomatosis with polyangiitis (GPA), microscopic polyangiitis (MPA), and eosinophilic granulomatosis with polyangiitis (EGPA), each presenting distinct clinical features [8,9].

Although ANCA antibodies are primarily present in AAV, they can also be useful in diagnosing other diseases. It is important to note that ANCA antibodies are associated with a heterogeneous group of rare autoimmune diseases that cause vasculitis with varying symptoms [10].

In our study, we focused solely on ANCA-positive small-vessel vasculitis, as the criteria for qualifying individual disease entities are precisely defined according to ACR–EULAR (American College of Rheumatology–European League Against Rheumatism) guidelines. The ACR–EULAR has recently introduced a new classification system. It take into account ANCA testing and modern imaging techniques, resulting in improved specificity and sensitivity. Therefore, in each case we were able to ascertain that the developed disease was associated with ANCA antibodies, confirmed by laboratory tests and biopsy [9,11]. We summarized the characteristics and clinical presentation of ANCA-positive small-vessel vasculitis cases reported in the literature since the beginning of the COVID-19 pandemic.

## 2. Materials and Methods

The research methods adhered to the PRISMA guidelines [12].

### 2.1. Search Strategy

**Figure 1:**
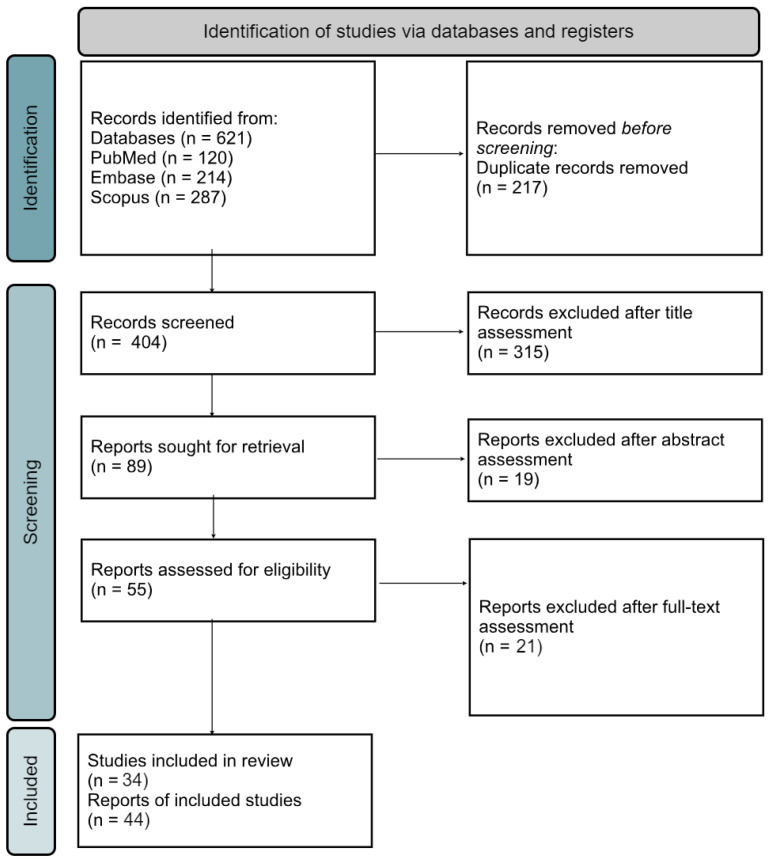
The PRISMA searching strategy flow chart.

A thorough systematic search was conducted, utilizing the Mendeley search engine, alongside screening of PubMed, Emboss, and Scopus databases. The scope of the research encompassed papers published from 1 January 2020 to 1 January 2024. Three researchers (K.L., A.W, and M.B.) independently conducted searches using a predefined set of keywords: (ANCA OR “antineutrophil cytoplasmic antibody” OR “ANCA-associated” OR Wegener OR granulomatosis OR “eosinophilic granulomatosis” OR “Churg-Strauss” OR microscopic OR “MPO-ANCA” OR “PR3-ANCA” OR MPO OR PR3) AND (vasculitis OR “granulomatosis polyangiitis” OR syndrome OR “de novo vasculitis” OR vasculitides) AND (COVID-19 OR “SARS-CoV-2” OR COVID) AND (Moderna OR Pfizer OR AstraZeneca OR Sputnik OR Janssen OR Sinopharm OR Novavax OR “CoronaVac” OR Covaxin OR Sanofi) AND (vaccination OR vaccine). Initially, 621 papers were identified, which underwent automatic exclusion of non-English articles and removal of duplicates, resulting in 404 papers for further analysis. Subsequently, the title, abstracts, and full texts of the remaining papers were meticulously reviewed by the three authors (K.L., A.W, M.B), with consensus reached on inclusion for evaluation. The comprehensive literature review yielded 34 papers, documenting 44 cases meeting the criteria for de novo ANCA-positive small-vessel vasculitis in temporal association with SARS-CoV-2 vaccination, as illustrated in Figure 1.

Data regarding patients’ metrics, comorbidities, vaccination characteristics, symptoms, diagnostics, treatment, and outcomes are summarized and depicted in Table 1, Table 2, Table 3, Table 4 and Table 5.

**Table 1:** New-onset ANCA-positive small-vessel vasculitis post-COVID-19 vaccine cases comparison. AAV-ANCA—associated vasculitis; ANCA—anti-neutrophil cytoplasmic antibody; COPD—chronic obstructive pulmonary disease; DM2—myotonic dystrophy type 2; EGPA—eosinophilic granulomatosis with polyangiitis; GERD—gastroesophageal reflux disease; HF—heart failure; HFrEF—heart failure with reduced ejection fraction; GPA—granulomatosis with polyangiitis; MPA—microscopic polyangiitis; MPO—myeloperoxidase; PR3–proteinase-3.

					Vaccination			
No.	Authors, date	Gender	Age	Comorbidities	Doses no.	1st	2nd	3rd
1.	Chan-Chung et al., 2022 [13]	F	62	asthma	2	Pfizer-BioNTech	Pfizer-BioNTech	-
2.	Hwang et al., 2023 [14]	F	71	chronic cough, diabetes	1	Pfizer-BioNTech	-	-
3.	Shirai et al., 2023 [15]	F	63	-	1	Pfizer-BioNTech	-	-
4.	Baier et al., 2022 [16]	F	57	-	3	Pfizer-BioNTech	Pfizer-BioNTech	Pfizer-BioNTech
5.	Ibrahim et al., 2022 [17]	F	79	asthma, GERD, sleep apnea	2	Moderna	Moderna	-
6.	Ting et al., 2023 [18]	F	63	asthma, bilateral cataract excision, macular degeneration	3	Pfizer-BioNTech	Pfizer-BioNTech	Pfizer-BioNTech
7.		M	51	HFrEF, hypertension	2	Pfizer-BioNTech	Pfizer-BioNTech	-
8.		M	62	diverticulosis, remote cholecystectomy, vitiligo	1	Pfizer-BioNTech	-	-
9.		F	70	DM2, dyslipidemia, hypertension, hypothyroidism	3	Moderna	Moderna	Moderna
10.	Alqatari et al., 2023 [19]	F	34	-	1	Pfizer-BioNTech	-	-
11.		F	61	-	2	Pfizer-BioNTech	Pfizer-BioNTech	-
12.		F	46	-	2	Pfizer-BioNTech	Pfizer-BioNTech	-
13.		F	46	hypothyroidism	1	Pfizer-BioNTech	-	-
14.		M	47	-	1	Pfizer-BioNTech	-	-
15.	Essien et al., 2022 [20]	M	27	-	1	Pfizer-BioNTech	-	-
16.	Al.Yafeai et al., 2022 [21]	F	62	Hashimoto’s disease	1	Pfizer-BioNTech	-	-
17.	Kim et al., 2022 [22]	F	72	-	3	Oxford AstraZeneca	Oxford AstraZeneca	Moderna
18.	Jha et al., 2023 [23]	M	51	-	1	Oxford AstraZeneca	-	-
29.	Gen et al., 2023 [24]	F	82	hypertension	3	Pfizer-BioNTech	Pfizer-BioNTech	Moderna
20.	Dourado et al., 2023 [25]	F	26	sinusitis	1	Pfizer-BioNTech	-	-
21.		M	47	-	2	Pfizer-BioNTech	Pfizer-BioNTech	-
22.	Prabhahar et al., 2022 [26]	M	51	-	1	Oxford AstraZeneca	-	-
23.	Yadav et al., 2022 [27]	F	52	hypertension	1	Janssen	-	-
24.	Zamoner et al., 2022 [28]	F	58	hyperthyroidism	1	Oxford AstraZeneca	-	-
25.	Christodoulou et al., 2022 [29]	F	72	-	2	Moderna	Moderna	-
26.	Hakroush et al., 2021 [30]	F	79	degenerative disc disease	2	Pfizer-BioNTech	Pfizer-BioNTech	-
27.	Chen et al., 2021 [31]	F	70	-	1	Moderna	-	-
28.	Saiz et al., 2023 [32]	F	48	-	2	Moderna	Moderna	Moderna
29.	Prema et al., 2021 [33]	M	58	-	2	Covaxin	Covaxin	-
30.		M	45	-	1	Covaxin	-	-
31.	Okuda et al., 2021 [34]	F	37	hyperthyroidism	1	Pfizer-BioNTech	-	-
32.	Mahdi et al., 2023 [35]	M	53	allergic rhinosinusitis, hypertension, chronic cough, COPD	3	Pfizer-BioNTech	Pfizer-BioNTech	-
33.	So et al., 2022 [36]	M	42	-	2	Pfizer-BioNTech	Pfizer-BioNTech	-
34.	De Cagna et al., 2023 [37]	M	21	asthma	2	Pfizer-BioNTech	Pfizer-BioNTech	-
35.	Loo et al., 2023 [38]	F	75	HF	1	Moderna	-	-
36.		M	59	-	1	Oxford AstraZeneca	-	-
37.	Moronti et al., 2023 [39]	M	67	hypertension	3	Oxford AstraZeneca	Moderna	Moderna
38.	Yoshino et al., 2023 [40]	M	56	gout	2	Pfizer-BioNTech	Pfizer-BioNTech	-
39.	Uddin et al., 2022 [41]	M	59	-	2	Pfizer-BioNTech	Pfizer-BioNTech	-
40.	Javadian et al., 2024 [42]	F	32	-	2	Sinopharm BIBP	Sinopharm BIBP	-
41.	Suzuki et al., 2022 [43]	M	72	prostatic hypertrophy	2	Pfizer-BioNTech	PFfizer-BioNTech	-
42.	Felzer et al., 2022 [44]	F	60	-	2	Moderna	Moderna	-
43.	Garcia et al., 2022 [45]	F	78	odontogenic maxillary sinusitis after sinus surgery	2	CoronaVac	CoronaVac	-
44.	Kawamura et al., 2023 [46]	F	71	hypertension	2	Pfizer-BioNTech	Pfizer-BioNTech	-

**Table 2:** New-onset ANCA-positive small-vessel vasculitis post-COVID-19 vaccine cases comparison. AAV-ANCA—associated vasculitis; ANCA—anti-neutrophil cytoplasmic antibody; COPD—chronic obstructive pulmonary disease; DM2—myotonic dystrophy type 2; EGPA—eosinophilic granulomatosis with polyangiitis; GERD—gastroesophageal reflux disease; HF—heart failure; HFrEF—heart failure with reduced ejection fraction; GPA—granulomatosis with polyangiitis; MPA—microscopic polyangiitis; MPO—myeloperoxidase; PR3–proteinase-3.

	Vaccination		Symptoms		
No.	Triggering dose	Vx to Sx days number	Inflammatory reaction	Respiratory sys.	Urinary sys.
1.	2	not specified	fever, weakness	-	edema
2.	1	2		-	-
3.	1	3	fever, headache	-	-
4.	3	1	-	dyspnea, pulmonary hemorrage	-
5.	2	14	weakness	-	edema
6.	1	not specified	-	-	renal impairment (increased creatinine level)
7.	1	17	-	-	renal impairment (increased creatinine level)
8.	1	24	-	-	renal impairment (increased creatinine level)
9.	3	42	fever, headache, weakness	-	renal impairment (increased creatinine level)
10.	1	7	-	pulmonary hemorrage	renal impairment (increased creatinine level)
11.	2	25	weakness	-	renal impairment (increased creatinine level)
12.	2	9	-	pulmonary hemorrage	-
13.	1	5	-	-	-
14.	1	20	-	-	renal impairment (increased creatinine level)
15.	1	4	fever, weakness	dyspnea	renal impairment (increased creatinine level)
16.	1	30	weakness,	dyspnea, haemoptysis, hematemesis, pulmonary hemorrage	renal impairment (increased creatinine level)
17.	1	not specified	anorexia	-	renal impairment (increased creatinine level)
18.	1	10	weakness	-	-
19.	3	1	fever, headache, weakness	hematemesis	renal impairment (increased creatinine level)
20.	1	14	fever	cough, haemoptysis, pulmonary hemorrage	renal impairment (increased creatinine level)
21.	2	90	anorexia, weakness	-	renal impairment (increased creatinine level)
22.	1	15	fever, weakness	-	renal impairment (increased creatinine level)
23.	1	12	fever, weakness	-	renal impairment (increased creatinine level)
24.	1	4	weakness	-	renal impairment (increased creatinine level)
25.	2	15	fever	dyspnea, hematemesis, pulmonary hemorrage	renal impairment (increased creatinine level)
26.	2	14	weakness		-
27.	1	7	weakness	dyspnea, haemoptysis, pulmonary hemorrage	edema, renal impairment (increased creatinine level)
28.	1	13	headache	dyspnea, haemoptysis	-
29.	2	14	-	dyspnea, haemoptysis, pulmonary hemorrage	renal impairment (increased creatinine level)
30.	1	12	-	dyspnea, haemoptysis, pulmonary hemorrage	edema, renal impairment (increased creatinine level)
31.	1	12	fever	-	renal impairment (increased creatinine level)
32.	3	7	weakness	-	edema
33.	2	0	weakness	dyspnea	edema, renal impairment (increased creatinine level)
34.	1	not specified	fever, weakness	dyspnea	edema, renal impairment (increased creatinine level)
35.	1	77	weakness	-	renal impairment (increased creatinine level)
36.	1	not specified	-	-	renal impairment (increased creatinine level)
37.	3	21	weakness	-	renal impairment (increased creatinine level)
38.	2	14	fever, weakness	-	renal impairment (increased creatinine level)
39.	2	12	fever, weakness	-	renal impairment (increased creatinine level)
40.	2	1	fever, weakness	dyspnea, haemoptysis	renal impairment (increased creatinine level)
41.	2	1	fever, weakness	-	renal impairment (increased creatinine level)
42.	1	not specified	anorexia, fever, headache, weakness	dyspnea	renal impairment (increased creatinine level)
43.	1	14	anorexia, fever, weakness	cough	renal impairment (increased creatinine level)
44.	2	7	fever, weakness	-	renal impairment (increased creatinine level)

**Table 3:** New-onset ANCA-positive small-vessel vasculitis post-COVID-19 vaccine cases comparison. AAV-ANCA—associated vasculitis; ANCA—anti-neutrophil cytoplasmic antibody; COPD—chronic obstructive pulmonary disease; DM2—myotonic dystrophy type 2; EGPA—eosinophilic granulomatosis with polyangiitis; GERD—gastroesophageal reflux disease; HF—heart failure; HFrEF—heart failure with reduced ejection fraction; GPA—granulomatosis with polyangiitis; MPA—microscopic polyangiitis; MPO—myeloperoxidase; PR3–proteinase-3.

	Symptoms				
No.	Nervous sys.	Musculoskeletal sys.	Integumentary sys.	Digestive sys.	Other
1.	extremities paresthesia	-	purpuric macules	-	-
2.	extremities paresthesia, peripheral neuropathy	myalgia	erythematous patch lesions	-	-
3.	hearing loss, vertigo	-	-	-	sinusitis, otitis media
4.	-	-	-	-	-
5.	extremities paresthesia	myalgia	-	-	-
6.	-	-	-	-	-
7.	-	-	-	-	-
8.	-	-	-	-	-
9.	-	-	-	-	-
10.	-	-	-	-	-
11.	-	-	-	-	-
12.	hearing loss	-	-	-	sinusitis
13.	hearing loss	-	-	-	sinusitis, otitis media
14.	-	-	-	-	sinusitis
15.	-	arthralgia, myalgia	purpuric macules	-	-
16.	-	arthralgia	-	-	-
17.	-	-	-	-	-
18.	extremities paresthesia	-	erythematous patch lesions, purpuric macules	-	-
19.	-	-	-	-	-
20.	-	-	-	-	-
21.	-	-	-	-	-
22.	-	arthralgia, synovitis	-	-	-
23.	-	arthralgia	-	-	-
24.	-	arthralgia	-	-	-
25.	-	-	-	-	-
26.	-	-	-	-	-
27.	-	-	-	-	-
28.	hearing loss, recurrent left facial paralysis	-	-	-	otitis media
29.	-	-	-	-	-
30.	-	-	-	-	-
31.	-	-	erythematous patch lesions, purpuric macules	-	-
32.	peripheral neuropathy	arthralgia, myalgia	purpuric macules	-	-
33.	-	-	-	-	-
34.	-	-	purpuric macules	vomiting	-
35.	vertigo	-	-	-	-
36.	-	-	-	-	-
37.	-	-	-	vomiting	-
38.	peripheral neuropathy	myalgia	-	vomiting	-
39.	-	arthralgia	-	-	-
40.	-	myalgia	-	-	-
41.	-	-	-	-	-
42.	extremities paresthesia, vertigo	myalgia	-	-	-
43.	-	-	-	-	-
44.	-	-	-	-	-

**Table 4:** New-onset ANCA-positive small-vessel vasculitis post-COVID-19 vaccine cases comparison. AAV-ANCA—associated vasculitis; ANCA—anti-neutrophil cytoplasmic antibody; COPD—chronic obstructive pulmonary disease; DM2—myotonic dystrophy type 2; EGPA—eosinophilic granulomatosis with polyangiitis; GERD—gastroesophageal reflux disease; HF—heart failure; HFrEF—heart failure with reduced ejection fraction; GPA—granulomatosis with polyangiitis; MPA—microscopic polyangiitis; MPO—myeloperoxidase; PR3–proteinase-3.

	Diagnostics		
No.	Biopsy findings	PR-3 (IU/mL)	MPO (IU/mL)
1.	heart: eosinophilic myocarditis without granulomas or giant cells, skin: small-vessel leukocytoclastic vasculitis	negative	positive (>200)
2.	skin: perivascular neutrophilic, eosinophilic, and some lymphocytic infiltration	negative	positive (>135)
3.	nasal mucosa: fibrin deposition in the small vessels and granulation tissue with intensive infiltration of inflammatory cells	positive (259)	negative
4.	biopsy not performed	positive (6.7)	positive (9.9)
5.	muscles and nerves: focal vasculitis involving small perimysial vessels, nerve vasculitis involving a small epineural vessel, eosinophils infiltration	negative	positive (103)
6.	kidney: fibrin/fibrinogen staining highlights crescents	negative	positive (54)
7.	kidney: fibrin/fibrinogen staining highlights crescents	negative	positive (105)
8.	kidney: fibrin/fibrinogen staining highlights crescents	negative	positive (145.4)
9.	kidney: segmental necrosis	negative	positive (>135)
10.	kidney: Pauci-immune crescentic GN	positive (N.S.)	negative
11.	kidney: Pauci-immune crescentic GN	positive (N.S.)	negative
12.	kidney: Pauci-immune crescentic GN	positive (N.S.)	negative
13.	kidney: Pauci-immune crescentic GN	positive (N.S.)	negative
14.	kidney: Pauci-immune crescentic GN	negative	positive (N.S.)
15.	kidney: Pauci-immune crescentic GN, segmental fibrinoid necrosis	positive (>100)	negative
16.	biopsy not performed	positive (>100)	negative
17.	kidney: Pauci-immune crescentic GN with fibrocellular crescents and global sclerosis in glomeruli and focal moderate tubular atrophy	negative	positive (>120)
18.	nerves: lymphohistocytic infiltration, hemosiderin deposition, vessel damage consistent with problable vasculitis	positive (N.S.)	negative
19.	kidney: Pauci-immune crescentic GN	negative	positive (296)
20.	biopsy not performed	positive (1610)	negative
21.	kidney: Pauci-immune necrotising crescentic GN	negative	positive (>134)
22.	kidney: Pauci-immune crescentic GN	positive (N.S.)	negative
23.	kidney: necrotizing and crescentic GN with insighnificant glomerular immune complex deposit	positive (N.S.)	positive (N.S.)
24.	kidney: crescentic GN with glomerular sclerosis, fibrous crescents, interstitial fibrosis, and tubular atrophy	negative	positive (N.S.)
25.	kidney: focal necrotizing GN with cellular crescents	negative	positive (20.3)
26.	kidney: Pauci-immune crescentic GN, severe acute tubular injury and interstitial nephritis, prominent eosinophilic infiltration, severe acute tubular injury with myoglobin casts	negative	positive (>134)
27.	kidney: Pauci-immune crescentic GN with acute severe renal vasculitis	negative	positive (470)
28.	inferior nasal turbinate: necrotizing epithelioid granuloma with vasculitis	positive (N.S)	negative
29.	kidney: linear staining for IgG on the GBM	positive (N.S.)	negative
30.	kidney: linear staining for IgG on the GBM	negative	positive (N.S.)
31.	skin: infiltrations of lymphocyte-dominated inflammatory cells around the capillaries in the dermis and subcutaneous fat	positive (28.3)	positive (494)
32.	kidney: Pauci-immune necrotising crescentic GN	negative	positive (>134)
33.	kidney: Pauci-immune crescentic GN	negative	positive (38.6)
34.	kidney: focal segmental necrotizing GN	negative	positive (740)
35.	kidney: crescentic GN without immunoglobulin deposition	negative	positive (>134)
36.	kidney: crescentic GN	negative	positive (57)
37.	kidney: intraglomerular thrombi, fibrinoid necrosis with neutrophilic granulocyte infiltrate and fibrocellular crescents, tubular atrophy and intratubular hyaline cylinders	negative	positive (394.9)
38.	kidney: Pauci-immune crescentic GN	negative	positive (1405)
39.	kidney: Pauci-immune crescentic GN	positive (69)	negative
40.	biopsy not performed	positive (127)	negative
41.	kidney: Pauci-immune crescentic GN	negative	positive (3150)
42.	lung: acute necrotizing granulomatous inflammation	positive (979)	negative
43.	kidney: necrotizing and crescentic GN	positive (19)	negative
44.	biopsy not performed	negative	positive (280)

**Table 5:** New-onset ANCA-positive small-vessel vasculitis post-COVID-19 vaccine cases comparison. AAV-ANCA—associated vasculitis; ANCA—anti-neutrophil cytoplasmic antibody; COPD—chronic obstructive pulmonary disease; DM2—myotonic dystrophy type 2; EGPA—eosinophilic granulomatosis with polyangiitis; GERD—gastroesophageal reflux disease; HF—heart failure; HFrEF—heart failure with reduced ejection fraction; GPA—granulomatosis with polyangiitis; MPA—microscopic polyangiitis; MPO—myeloperoxidase; PR3–proteinase-3.

	Treatment	
No.	Treatment	Outcome
1.	steroids, RTX, gabapentin	recovery
2.	steroids, CYC	recovery; leukopenia after CYC achieving remission, at 200 days follow-up patient still receiving prednisolone
3.	steroids, CYC	recovery
4.	steroids, PEX	recovery
5.	steroids, AZA	recovery
6.	steroids, CYC, PEX, AZA	recovery
7.	steroids, CYC, PEX, dialysis	partial recovery; at 11 months follow-up patient still dyalisis-dependent with no evidence of renal recovery
8.	steroids, CYC, PEX, dialysis	death due to multiorgan failure; patient dialysis-dependent at time of death
9.	steroids, CYC, PEX, dialysis	recovery
10.	steroids, RTX, PEX	recovery
11.	steroids, CYC, PEX, dialysis	death
12.	steroids, RTX	recovery
13.	steroids, CYC	recovery
14.	steroids, dialysis	no recovery; no further data
15.	steroids, RTX, losartan	recovery
16.	steroids, CYC, RTX, PEX, dialysis, IVIG, antimicrobials	partial recovery; improvement in pulmonary functions, no improvement in neurological functions
17.	steroids, CYC, PEX	recovery
18.	steroids, CYC	partial recovery; at 6 month follow-up patient self-ambulant with persistent distal upper and lower limb weakness
19.	steroids	recovery
20.	steroids, CYC, RTX	recovery; at the time of follow-up patient still receiving MTX and prednisolone
21.	steroids, CYC, RTX, PEX, dialysis	partial recovery; at the time of follow up patient still dialysis-dependent and receiving prednisolone
22.	steroids, RTX	recovery
23.	steroids, CYC, NSAIDs, antibiotics	partial recovery; discharged on CYC due to lack of improvement of renal function; final treatment outcome unknown
24.	steroids, CYC, AZA	partial recovery; at the time of follow-up still on pharmacotheraphy with increased creatinine levels
25.	steroids, CYC, PEX	recovery
26.	steroids, CYC	recovery
27	steroids, PEX, anti-CD20 therapy	recovery
28.	steroids, RTX, PPIs, MTX,	no recovery; no improvement in renal functions, no hearing improvement, breathing difficulties
29.	steroids, CYC, PEX, dialysis	recovery
30.	steroids, CYC, PEX, dialysis	recovery
31.	steroids	recovery
32.	steroids, CYC, RTX	recovery
33.	steroids, RTX, PEX	recovery
34.	steroids, RTX	recovery
35.	steroids, RTX, dialysis	recovery; dialysis-independent at the time of follow-up
36.	steroids, RTX, PEX	recovery
37.	steroids, PEX, dialysis, eculizumab	partial recovery; dialysis-dependent at 3 months after discharge
38.	steroids, CYC, RTX, MTX	recovery
39.	steroids, RTX	recovery
40.	PEX, dialysis, tocilizumab	partial recovery; dialysis-independent, on pharmacotherapy for CKD at the time of follow-up
41.	steroids, RTX, dialysis	recovery
42.	steroids, RTX	partial recovery;
43.	steroids, CYC, dialysis, MMF	partial recovery; at the time of the follow-up still receiving MMF and steroids, on pharmacotherapy for CKD
44.	steroids, CYC, AZA	recovery; positive HLA-DRB1*09:01 allele

GBM—glomerular basement membrane; GN—glomerulonephritis; AZA—azathioprine; CKD—chronic kidney disease; CYC—cyclophosphamide; IVIG—intravenous immunoglobulin; MMF—mycophenolate mofetil; MTX—methotrexate; NSAIDs—nonsteroidal anti-inflammatory drugs; PEX—plasma exchange; PPIs—proton-pump inhibitors; RTX—rituximab.

### 2.2. Criteria for Inclusion and Exclusion

The inclusion criteria encompassed all case reports published in English, involving patients aged 18 years or older, exhibiting de novo ANCA-vasculitis subsequent to COVID-19 vaccination, and confirmed through laboratory testing for AAV. Conversely, exclusion criteria comprised cases of AAV following SARS-CoV-2 infection, AAV relapses, manifestations of the disease lacking ANCA positivity, undocumented or untested ANCA status, and confirmed history of COVID-19 infection.

## 3. Results

**Figure 2:**
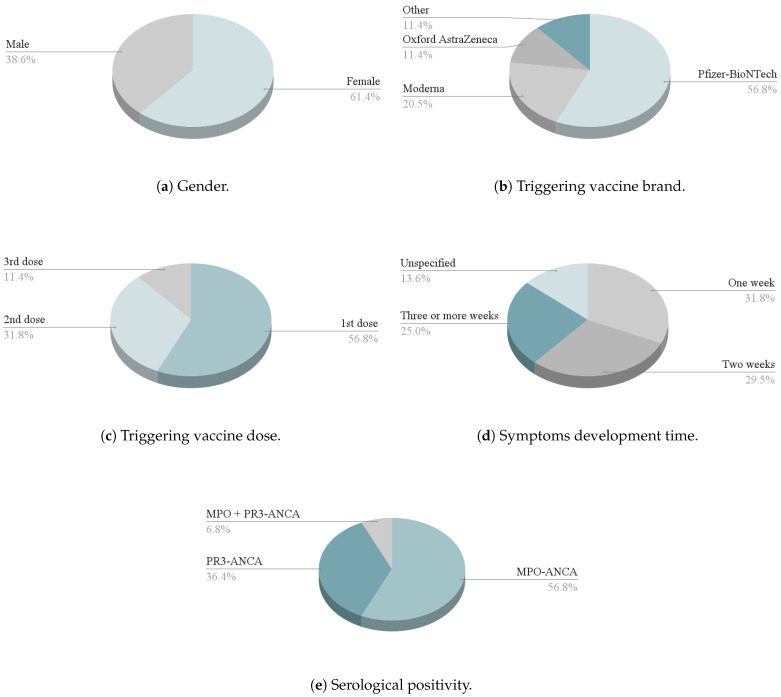
Charts present proportions of selected results.

**Figure 3:**
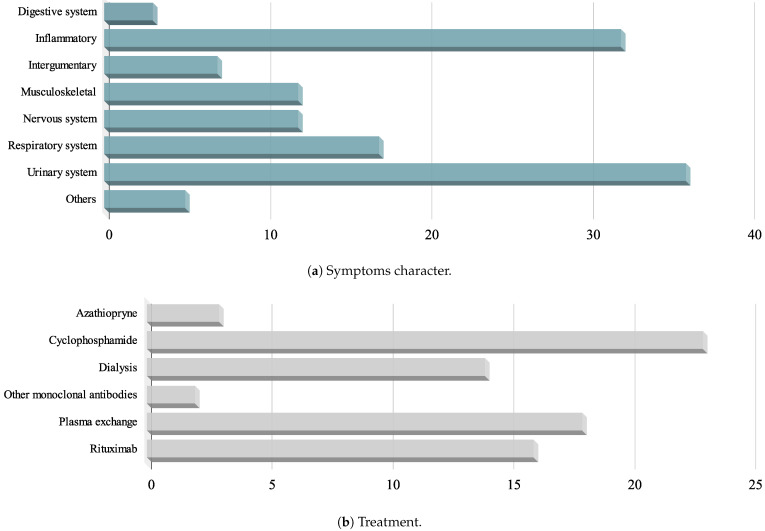
Charts presenting the contributions of individual systems in symptoms manifestation (**a**) and treatment used (**b**).

After conducting a thorough examination of the full text, 34 studies encompassing 44 patients were incorporated into the analysis. Percentages were derived from the number of patients who reported specific parameters. Acquired data are graphically presented in Figure 2 and Figure 3.

### 3.1. Demographics and Clinical Characteristics

The cohort was primarily composed of females, accounting for 27 out of 44 patients (61.4%). Age data were consistently documented across all cases, revealing a mean age of 57 (SD 15.09). Prior medical history and comorbidity information were available for 23 cases. The most common comorbidities included hypertension (n = 7, 16.0%), asthma (n = 4, 9.1%), hypothyroidism (n = 3, 6.8%), and hyperthyroidism (n = 2, 4.5%).

### 3.2. Vaccination Characteristics

Among patients diagnosed with de novo AAV, 25 (56.8%) received the Pfizer-BioNTech vaccine, 9 (20.5%) received Moderna, 5 (11.4%) received Oxford AstraZeneca, and 5 (11.4%) received other vaccines (Janssen, Covaxin, CoronaVac, Sinopharm BIBP). Most patients received either two doses of vaccine (n = 19, 43.2%) or one dose (n = 18, 40.9%). The remaining patients were administered three doses (n = 7, 15.9%). AAV was triggered in 25 (56.8%) cases following the first vaccine dose and in 14 (31.8%) patients after the second dose. Symptoms manifested after the third vaccine dose in 5 patients (11.4%).

### 3.3. Clinical Manifestations

Symptoms emerged within 1 week in 14 patients (31.8%), within 2 weeks in 13 cases (29.5%), and within 3 or more weeks in 11 cases (25.0%). In 6 cases (13.6%), the duration from vaccination to symptom onset was unspecified.

The most frequent manifestations were associated with inflammatory reactions (n = 32, 72.7%), urinary tract issues (n = 36, 81.8%), and respiratory manifestations (n = 17, 38.6%).

Signs of inflammatory reactions were observed in 32 cases. Among them, weakness was reported in 26 patients (81.3%), fever in 18 patients (56.3%), and headache in 5 patients (15.6%).

Urinary tract issues were observed in 36 patients. Among them, renal impairment was noted in 33 cases (91.7%), and edema was reported in 6 cases (16.7%).

The most prevalent respiratory manifestations included dyspnea (n = 12, 70.6%), pulmonary hemorrhage (n = 9, 53%), and haemoptysis (n = 7, 41.2%).

Symptoms pertaining to the nervous system manifested in 12 patients (26.7%), encompassing extremities paresthesia (n = 5, 41.7%), hearing loss (n = 4, 33.3%), peripheral neuropathy (n = 3, 25%), and vertigo (n = 3, 25%). Additionally, one case exhibited recurrent left facial paralysis.

Dermatological symptoms were evident in 7 patients (15.6%), including purpuric macules (n = 6, 85.7%) and erythematous patch lesions (n = 3, 42.9%).

Musculoskeletal manifestations were present in 12 cases (27.3%), with myalgia reported in 7 patients (58.3%) and arthralgia in 4 (33.3%).

Digestive tract symptoms were also observed in 3 cases (6.8%), manifested by vomiting.

### 3.4. Diagnostics

The ANCA antibody status was documented in 44 cases, all yielding positive serology. Among instances of de novo AAV post-SARS-CoV-2 vaccination, 25 (56.8%) were positive for MPO-ANCA, with 16 (36.7%) cases exhibiting PR3-ANCA positivity. Additionally, 3 (6.8%) cases showed dual positivity for MPO- and PR3-ANCA. Biopsy was employed to confirm AAV in 39 cases (88.6%), while it was not conducted in 5 cases. The most frequently reported finding in kidney biopsy was Pauci-immune crescentic glomerulonephritis, observed in 18 cases (40.9%).

### 3.5. Treatment

All AAV cases, except for one patient, received glucocorticoid therapy as the initial treatment. Rituximab, a chimeric monoclonal antibody targeted against CD20 on B cell surfaces, was administered to 16 patients (36.4%), while cyclophosphamide was utilized in 23 patients (52.3%) for further treatment. Additional therapeutic plasma exchange (PEX) was required in 18 patients (40.9%). Dialysis was deemed necessary for 14 cases (31.8%). Azathioprine was employed in only 3 cases (6.8%). Other monoclonal antibodies (benralizumab, eculizumab, tocilizumab) were utilized in 2 cases (4.5%).

### 3.6. Outcome

The outcome has been reported in all cases (n = 44). Recovery has been achieved in 30 cases (68.2%), while partial recovery was observed in 10 cases (22.7%). Additionally, 6 patients (13.6%) were dialysis-independent at the time of follow-up. Fatalities have been reported in 2 out of 44 cases (4.5%).

## 4. Discussion

### 4.1. Clinical Characteristics

In this study, we present a comprehensive analysis of de novo ANCA-positive small-vessel vasculitis onset following COVID-19 vaccination. In our cohort, females accounted for the majority of reported cases (61.4%), contrasting with the global prevalence. However, the mean age of 57 years was consistent with global trends [47]. Among the analyzed patients diagnosed with EGPA, a majority (60.0%) had a history of asthma. Prior studies have strongly associated asthma with EGPA, as a crucial prodromal syndrome often manifesting many years before the active vasculitis phase. In patients with persistent asthma that is difficult to control, consideration should be given to the possibility of EGPA diagnosis [48,49,50]. According to ACR/EULAR criteria from 2022, clinical and laboratory criteria for EGPA assign obstructive airway disease and nasal polyps, respectively, +3 points each, and blood eosinophil count $⩾1\times 10^{9}$/L for +5 points, which strongly correlates with symptoms of asthma.

### 4.2. Vaccines

The administration of vaccinations and the number of doses among the analyzed patients aligns with previous global data, as per the distribution reported by the European Union [51]. More than half of the patients developed their first symptoms after the first dose, and over one-third experienced symptoms after the second dose. This suggests that new-onsets of AAV may occur more frequently after the initial vaccination protocol rather than booster doses. The relationship between vaccines and autoimmune reactions is well-documented in the literature, with several theories proposed. Molecular mimicry, defined as the structural similarity between certain human proteins and antigens present in the vaccine, can lead to cross-reactions. In this scenario, the immune system may recognize human proteins as pathogens in the vaccines [2]. It has been established that the SARS-CoV-2 spike glycoprotein exhibits mimicry with certain human proteins such as alveolar lung surfactant proteins [52]. Adjuvants, included in vaccines to enhance and prolong the immune response, may also contribute significantly to triggering vaccine-related autoimmunity by activating cells of the innate immune system [2]. This emphasizes the importance of considering autoimmune disease after vaccination in the differential diagnosis. Despite that, vaccination remains a crucial tool during the pandemic and should not be withheld or restricted.

The cause of autoimmune diseases remains unclear, but genetic, immunological, hormonal, and environmental factors are believed to be significant triggers. Typically, autoimmunity does not lead to clinical symptoms unless another event, such as exposure to an environmental factor, prompts overt expression [53]. While there are certain theories regarding vaccine-related autoimmunity mechanisms, further studies are necessary. Nonetheless, in predisposed cases, COVID-19 vaccines may indeed trigger the onset of de novo AAV [54,55].

### 4.3. Clinical Presentation

Based on clinical presentation, antineutrophil cytoplasmic antibody-associated vasculitis encompasses three primary diseases: granulomatosis with polyangiitis, eosinophilic granulomatosis with polyangiitis, and microscopic polyangiitis. The clinical spectrum of AAV is extensive, resulting in diverse presentations, ranging from a skin rash to severe, life-threatening multisystem disease [56]. However, it most frequently affects the upper airways, lungs, kidneys, nervous system, and eyes [57].

The initial manifestations of AAV in most patients occurred within 1 to 3 weeks after vaccination. In our cases, weakness, fever, and headache, accompanied by inflammatory reactions, were the most frequent presentations. AAV may begin and present with constitutional symptoms suggestive of chronic inflammatory disease (fatigue, weight loss, fever, night sweats, myalgia, or polyarthralgia) [58]. It can complicate the initial clinical approach to the patient and early identification of the disease, leading to delay in diagnosis and treatment [59].

In our cases, a notable occurrence of dyspnea, pulmonary hemorrhage, and haemoptysis was observed, particularly among patients diagnosed with GPA and EGPA. Additionally, all patients diagnosed with GPA exhibited symptoms of sinusitis, consistent with the characteristic granulomatous lesions of the upper and lower respiratory tract often seen in GPA patients [60]. Lower respiratory tract involvement is a prevailing clinical characteristic, occurring in 45% to 67% of individuals diagnosed with ANCA-associated vasculitis, as indicated by studies [57,59]. The incidence of pulmonary involvement is higher in patients with granulomatosis with polyangiitis (GPA) and eosinophilic granulomatosis with polyangiitis (EGPA) (85–90%) than in patients with microscopic polyangiitis (MPA) (25–60%) [61].

Renal involvement is another leading clinical feature of ANCA-associated vasculitis, occurring in approximately 50% to 65% of patients [62]. We noted a significant prevalence of renal symptoms, elevated creatinine levels and extremity edema, particularly among patients diagnosed with GPA and MPA. Kidney involvement was confirmed through organ biopsy, revealing Pauci-immune crescentic glomerulonephritis in the majority of cases. According to the 2022 ACR/EULAR Classification Criteria for AAV, Pauci-immune glomerulonephritis serves as a histological criterion for both MPA and GPA, with a scoring range of +3 for MPA and +2 for GPA [11].

Approximately 30% of patients with ANCA-associated vasculitis may clinically exhibit symptoms such as muscle weakness, difficulty walking, numbness, and peripheral neuropathy [57,63]. Among our patients, symptoms related to the nervous and musculoskeletal systems were also noted. Predominantly, patients exhibited extremities paresthesia, hearing loss, myalgia, and arthralgia.

Dermatologic features may manifest in fewer than 20% of patients with ANCA-associated vasculitis [64]. Purpuric macules and erythematous patch lesions were the most characteristic presentations observed in the cases. However, cutaneous manifestations observed in AAV may exhibit varying clinical presentations among patients with GPA, MPA, and EGPA [65].

### 4.4. Serological Diagnostics

ANCAs, a class of autoantibodies primarily of the IgG type, target antigens located in the cytoplasmic granules of polymorphonuclear neutrophil granulocytes (PMNs). They are notably linked with a category of disorders termed ANCA-associated vasculitis, characterized by systemic vasculitis. There are two major types of ANCAs distinguished by their cellular localization patterns: one type associated with perinuclear staining (p-ANCA), and the other type associated with diffuse cytoplasmic staining (c-ANCA) [66].

c-ANCAs, which specifically react with PR3, are a primary laboratory marker for GPA and are present in 80–90% of patients, with their titer correlating with disease activity. They are also found in MPA, EGPA, and Pauci-immune crescendic glomerulonephritis (25–36%). On the other side, p-ANCAs, that are specifically reacting with MPO, are found in all AAVs, but also in drug-induced immune reactions. Non-specifically reacting with MPO also may occur in the course of non-specific inflammatory bowel diseases (in 60–70% of patients with ulcerative colitis and 10% of individuals with Crohn’s disease), autoimmune liver diseases (in 90% of patients with primary sclerosing cholangitis and 70% of patients with autoimmune hepatitis), and HIV infections (18%), as well as in patients with connective tissue diseases (in 20–25% of patients with systemic lupus erythematosus, 20% of patients with mixed connective tissue disease) [10,67,68].

Therefore, conducting a comprehensive evaluation is crucial for confirming the diagnosis of AAV, excluding other potential causes, and evaluating the severity of the disease and its impact on organs. Typically, diagnosis involves a combination of clinical evaluation, serological assays, radiological findings, and histopathological analyses [69].

### 4.5. Treatment and Outcome

Treatment for antineutrophil cytoplasmic autoantibody-associated vasculitis in most patients involved a combination of immunosuppressive medications to control inflammation and prevent further damage to affected organs. The first-line drugs among these patients were glucocorticoids (GC), mostly combined with rituximab (RTX) or cyclophosphamide (CYC). Such therapeutic management corresponds to the updated guidelines on the management of AAV released in 2022 by EULAR. According to EULAR, the gold standard of inducing remission in new-onset patients with active GPA or MPA comprises glucocorticoids with taper after 4–5 months or avacopan supply combined with RTX (or methotrexate (MTX) or mycophenolate mofetil (MMF)) in not organ-/life-threatening cases and combined with RTX or CYC in organ-/life-threatening cases. In new-onset EGPA with organ-/life-threatening symptoms, it is recommended to treat with a combination of high-dose GCs and CYC. However, RTX may be considered as an alternative instead of CYC [70]. The choice of therapy should be based on the patient’s individual characteristics and clinical condition, with the assumption that RTX is favored over CYC in children and adolescents, pre-menopausal women and men, frail elderly, glucocorticoid-sparing and PR3-ANCA. Preference for RTX over CYC in patients of reproductive age stems from concerns about the long-term safety of CYC according to fertility and preserving reproductive potential. Cyclophosphamide can lead to premature ovarian failure due to a reduction of ovarian reserve and also might increase the risk of male infertility [71]. Additionally, there is evidence that CYC has mutagenic effects and may contribute to cancers, including acute leukaemia, bladder and urinary tract cancers, and other malignant neoplasms [72,73]. Our cohort consists of 14 patients of reproductive age (15–49), 8 females and 6 males; 11 of them were treated by RTX or/and CYC administration. At the same time, the majority of them took RTX, which is recommended for reproductive patients. Cyclophosphamide, if given, was taken by elderly patients. Additionally, the efficacy of combining RTX with CYC in cases of AVV with serum creatinine >350 μmol/L is under investigation [74]. Five patients received combined CYC and RTX therapy, with renal impairment expressed by increased serum creatinine level among four of them. According to treatment recommendations, patients with organ-/life-threatening type with rapidly progressive glomerulonephritis should be evaluated for the use of plasmapheresis [70]. Among the presented patients, plasmapheresis was used in 18 cases, all with life-threatening symptoms, such as pulmonary hemorrhage and/or renal impairment. For maintenance of remission of GPA and MPA, treatment with RTX is recommended after induction of remission with RTX or CYC. AZA or MTX could also be considered as an alternative. According to recommendations for the maintenance of remission of EGPA, AZA, MTX, mepolizumab, or RTX with GCs taper are suggested. All of the above correspond with the presented treatment strategies in our review. Recent studies have highlighted the beneficial effects of avacopan, a complement receptor C5a inhibitor, in the treatment of renal lesions in AAV patients. It is characterized by a more effective reduction of albuminuria and a lower percentage of serious adverse effects, including a lower rate of white blood cell decline and the risk of severe infections compared with prednisolone [70,75]. As avacopan was included in EULAR recommendations as a suggested first-line drug in the treatment of AAV alongside GCs, given its favorable impact on both remission but also fewer adverse effects, it should have been considered as a drug of choice among clinicians [70]. With a combination of immunosuppressive medications, remission rates have notably increased over the years. The prompt initiation of therapy is crucial to prevent severe organ damage. Close monitoring and regular follow-ups are integral to managing the disease and reducing the risk of relapse.

There is insufficient evidence to establish causality, whether the vaccine triggered the reaction, revealed an underlying condition, or if it was coincidental. The patient’s symptoms and deterioration began after receiving the vaccine, raising concerns about a possible connection. However, in southern Japan, an increase in new-onset AAV cases was observed following the initiation of the COVID-19 vaccination program [76]. Nonetheless, a recent study suggests no significant relationship exists between COVID-19 vaccination and new AAV onsets [77].

## 5. Limitations

Our study is subject to limitations, with the most important being that the data for our analysis was often incomplete, with not every parameter described. We attempted to select and analyze only the most frequently reported factors. The majority of papers did not provide information about the race of the patients. As a result, we were unable to compare the frequency of occurrence of various AAVs across different continents. In addition, we observed a lack of precise timing of the initial manifestation of symptoms, and in some cases this required calculating the average time based on a range of weeks. Laboratory parameters varied across different laboratories; therefore, we could not compare the antibody titer of ANCA antibodies. Moreover, in most cases only MPO and PR3 antibodies were included in the diagnostic. Currently, AAV diagnostic uses panels containing other antibodies (bactericidal permeability-increasing protein (BPI), lactoferrin, cathepsin G, lysozyme, and elastase) [78]. Their use in further work would translate into greater systematization of results and more accurate conclusions about patients with post-COVID-19 vasculitis.

Due to the rarity of AAV-new-onsets after COVID-19 vaccination and the retrospective nature of the events, it is not possible to estimate the relative risk of these adverse events.

## 6. Conclusions

This systematic review provides the most recent summary of reported post-COVID-19 vaccine new-onset of ANCA-positive small-vessel vasculitis. We present a comprehensive résumé, with the potential to serve as a reliable source of knowledge on that topic.

Although vaccination is a pillar of the fight against infectious diseases, it should be emphasized that the body on the path of overreaction to vaccination can lead to the development of autoimmune diseases. For this reason, observation of susceptible individuals with even mild post-vaccination symptoms suggesting AAV is crucial to prevent life-threatening conditions.

## Data Availability

No new data were created or analyzed in this study. Data sharing is not applicable to this article.
